# Diagnostic performance of CT scan–based radiomics for prediction of lymph node metastasis in gastric cancer: a systematic review and meta-analysis

**DOI:** 10.3389/fonc.2023.1185663

**Published:** 2023-10-23

**Authors:** Zanyar HajiEsmailPoor, Peyman Tabnak, Behzad Baradaran, Fariba Pashazadeh, Leili Aghebati-Maleki

**Affiliations:** ^1^ Faculty of Medicine, Tabriz University of Medical Sciences, Tabriz, Iran; ^2^ Immunology Research Center, Tabriz University of Medical Sciences, Tabriz, Iran; ^3^ Department of Immunology, Faculty of Medicine, Tabriz University of Medical Sciences, Tabriz, Iran; ^4^ Research Center for Evidence-based Medicine, Iranian Evidence-Based Medicine (EBM) Centre: A Joanna Briggs Institute (JBI) Centre of Excellence, Faculty of Medicine, Tabriz University of Medical Sciences, Tabriz, Iran

**Keywords:** radiomics, machine learning, artificial intelligence, lymph node metastasis, gastric cancer

## Abstract

**Objective:**

The purpose of this study was to evaluate the diagnostic performance of computed tomography (CT) scan–based radiomics in prediction of lymph node metastasis (LNM) in gastric cancer (GC) patients.

**Methods:**

PubMed, Embase, Web of Science, and Cochrane Library databases were searched for original studies published until 10 November 2022, and the studies satisfying the inclusion criteria were included. Characteristics of included studies and radiomics approach and data for constructing 2 × 2 tables were extracted. The radiomics quality score (RQS) and Quality Assessment of Diagnostic Accuracy Studies (QUADAS-2) were utilized for the quality assessment of included studies. Overall sensitivity, specificity, diagnostic odds ratio (DOR), and area under the curve (AUC) were calculated to assess diagnostic accuracy. The subgroup analysis and Spearman’s correlation coefficient was done for exploration of heterogeneity sources.

**Results:**

Fifteen studies with 7,010 GC patients were included. We conducted analyses on both radiomics signature and combined (based on signature and clinical features) models. The pooled sensitivity, specificity, DOR, and AUC of radiomics models compared to combined models were 0.75 (95% CI, 0.67–0.82) versus 0.81 (95% CI, 0.75–0.86), 0.80 (95% CI, 0.73–0.86) versus 0.85 (95% CI, 0.79–0.89), 13 (95% CI, 7–23) versus 23 (95% CI, 13–42), and 0.85 (95% CI, 0.81–0.86) versus 0.90 (95% CI, 0.87–0.92), respectively. The meta-analysis indicated a significant heterogeneity among studies. The subgroup analysis revealed that arterial phase CT scan, tumoral and nodal regions of interest (ROIs), automatic segmentation, and two-dimensional (2D) ROI could improve diagnostic accuracy compared to venous phase CT scan, tumoral-only ROI, manual segmentation, and 3D ROI, respectively. Overall, the quality of studies was quite acceptable based on both QUADAS-2 and RQS tools.

**Conclusion:**

CT scan–based radiomics approach has a promising potential for the prediction of LNM in GC patients preoperatively as a non-invasive diagnostic tool. Methodological heterogeneity is the main limitation of the included studies.

**Systematic review registration:**

https://www.crd.york.ac.uk/Prospero/display_record.php?RecordID=287676, identifier CRD42022287676.

## 1 Introduction

Despite advancements in identification and treatment, gastric cancer (GC) remains a significant global health challenge, ranking as the fifth most diagnosed cancer globally and the fourth leading cause of cancer-related mortality, with an estimated 769,000 deaths reported in 2020 alone (1). The selection of the optimal treatment strategy for GC is largely based on the tumor-nodal-metastasis (TNM) staging system, which assesses the extent of tumor invasion through the different layers of the stomach (T), lymph node involvement (N), and distant metastasis (M). This staging system is important in determining the most appropriate treatment approach, such as surgery, chemotherapy, and/or radiation therapy, and has been shown to be a reliable predictor of patient outcomes (2). Accurate determination of lymph node metastasis (LNM) status is critical for optimal management of GC. As the main component of TNM staging, LNM status is used to select the appropriate preoperative treatment strategy and is also an important prognostic factor for patient survival and tumor recurrence after surgical resection. Thus, it is essential to accurately determine LNM status (3, 4). Current traditional imaging methods for assessing nodal status are based on lymph node (LN) shape, enhancement, and size, which can be normal or enlarged. Most patients may be misclassified for nodal staging in the TNM system. To date, computed tomography (CT) is the most common imaging modality, which is widely used for preoperative estimation of nodal status. However, the reported overall accuracy was low and unsatisfactory. Therefore, it is necessary to establish more precise methods to supplement the current methods of assessing LN status (5–7).

Recently, radiomics has attracted more attention as the methodology of translating medical images into reproducible and quantitative data for clinical decision support. Radiomics extracts quantitative features, so-called *radiomics features*, from diagnostic images by using mathematical machine learning or deep learning algorithms to uncover the hidden tumor characteristic, which is not seen by the naked eye and helps predict the considered outcome, for example, LNM prediction. In detail, radiomics features are extracted from the region of interest (ROI) or volume of interest (VOI). When two-dimensional (2D) ROI or (3D) VOI is delineated by a radiologist, software, or both (*image segmentation*), the different types of radiomics features (e.g., histogram based and texture based) are extracted by mathematical methods. Maybe hundreds of radiomics features are extracted; however, most of them are redundant and non-informative. Therefore, they have to be transformed or removed (*dimensionality reduction*), and then the most informative features should be selected (*feature selection*). Finally, a predictive model is established based on the selected features (*model construction*) to predict the outcome (e.g., LNM prediction) (8, 9). Hence, radiomics can capture a lot of valuable invisible information non-invasively and more precisely.

In this meta-analysis, we have collected evidence from previous studies to further investigate the diagnostic accuracy of CT-based radiomics for predicting LNM metastasis status in GC patients in order to help applying the radiomics approach in clinical practice.

## 2 Materials and methods

This systematic review and meta-analysis were conducted according to the recommendations of the Preferred Reporting Items for Systematic Reviews and Meta-Analyses (PRISMA) guidelines (**Supplementary Material**) (10). The study protocol was registered on the International Prospective Register of Systematic Reviews (PROSPERO) prospectively (registration no. CRD42022287676).

### 2.1 Literature search

A computerized search of PubMed, Embase, Web of Science, and Cochrane Library databases was performed without a limitation of a start date for studies published until 16 August 2022. We searched databases for the second time on 10 November 2022 to discover newly published studies. All related search terms and synonyms were considered in the search strategy as follows: [(GC) OR (gastric tumor) OR (stomach cancer) OR (stomach tumor)] AND [(CT) OR (computed tomography)] AND [(lymph node) OR (lymphatic) OR (lymphovascular)] AND [(radiomic) OR (radiomics) OR (texture)]. We used Mendeley software, version 1.19.8, and Rayyan (11) for managing references. Two observers (Z.H. and P.T.) screened references by title and abstract to determine eligibility. Then, the full text based on inclusion and exclusion criteria was reviewed. Also, included study references were manually searched to find additional eligible studies. We restricted the search to the studies published in English. Uncertainties were resolved by consulting the third observer (L.A.M.).

### 2.2 Inclusion criteria

We selected studies satisfying the following PICO criteria: (1) population: patients diagnosed with GC; (2) index test: index test used CT scan for detection of LNM; (3) comparator test: for comparison, histopathologic results were considered as the reference standard; and (4) test accuracy or outcome: studies provided the area under the curve (AUC), sensitivity, and specificity data of CT-based radiomics or the corresponding data for a 2 × 2 contingency table construction.

### 2.3 Exclusion criteria

Exclusion criteria were set as follows: (1) studies in the form of conference abstracts, review articles, case reports, editorial, comments, letters, and animal studies; (2) studies not related to the CT scan–based radiomic prediction of LNM or GC; (3) studies in languages other than English; and (4) unable to construct 2 × 2 contingency table.

### 2.4 Data extraction

The following data were extracted, regarding patient, study, and CT-based radiomics characteristics using a standardized table: (1) patient characteristics: patients sample size, training, and testing group population sample size, patients sex numbers, mean age, and numbers of recruitment center number; (2) study characteristics: study origin (first author and country), publication year, study design, CT scan data, reference standard, and positive LNM ratio; (3) radiomics characteristics: image segmentation information, model features selection, and extraction methods, model or nomogram construction methods.

### 2.5 Quality assessment

The methodological quality of included studies was assessed using the Quality Assessment of Diagnostic Accuracy Studies 2 (QUADAS-2) (12) tool and radiomics quality score (RQS) (13). Two independent observers (Z.H. and P.T.) conducted data extraction and quality assessment. Any disagreement was resolved by reaching a consensus.

### 2.6 Statistical analysis

This meta-analysis was performed on MIDAS module in STATA 14.0 (StataCorp, Texas, United States). We quantified predictive accuracy by calculating pooled sensitivity, specificity, diagnostic odds ratio (DOR), positive likelihood ratio (PLR), and negative likelihood ratio (NLR) with 95% confidence interval (CI). The summary receiver operating characteristic curve (SROC) was created, and AUCs were used to summarize diagnostic accuracy. *I* ^2^ values were calculated to assess statistical heterogeneity among the included studies. *I* ^2^ values of 0%–25%, 25%–50%, 50%–75%, and > 75% represent very low, low, medium, and high-statistical heterogeneity, respectively. Coupled forest plots were created for showing pooled sensitivity and specificity. Studies and effect sizes were pooled using a random-effect model, indicating that the estimation of the distribution of true effects between studies considers heterogeneity. The presence of threshold effects was investigated in MetaDisc 1.4 by computing the Spearman’s correlation coefficient (*r*) between the logit (true positive rate) and logit (false positive rate). Subgroup analysis was performed to investigate the heterogeneity causes. The following covariates were selected to assess which factor causes heterogeneity: top left method used or not, segmentation dimension, arterial or venous phase of CT scan, tumoral or nodal segmentation, and automatic or manual segmentation. Furthermore, to assess the impact of included studies on the overall estimate, a sensitivity analysis was performed by eliminating each study. Deeks’ funnel plot was created to examine publication bias. Some studies did not report sensitivity and specificity to construct 2 × 2 table construction. Thus, we used the receiver operating curve (ROC) to calculate sensitivity and specificity using the top left method (14).

## 3 Results

### 3.1 Literature search

**Figure 1:**
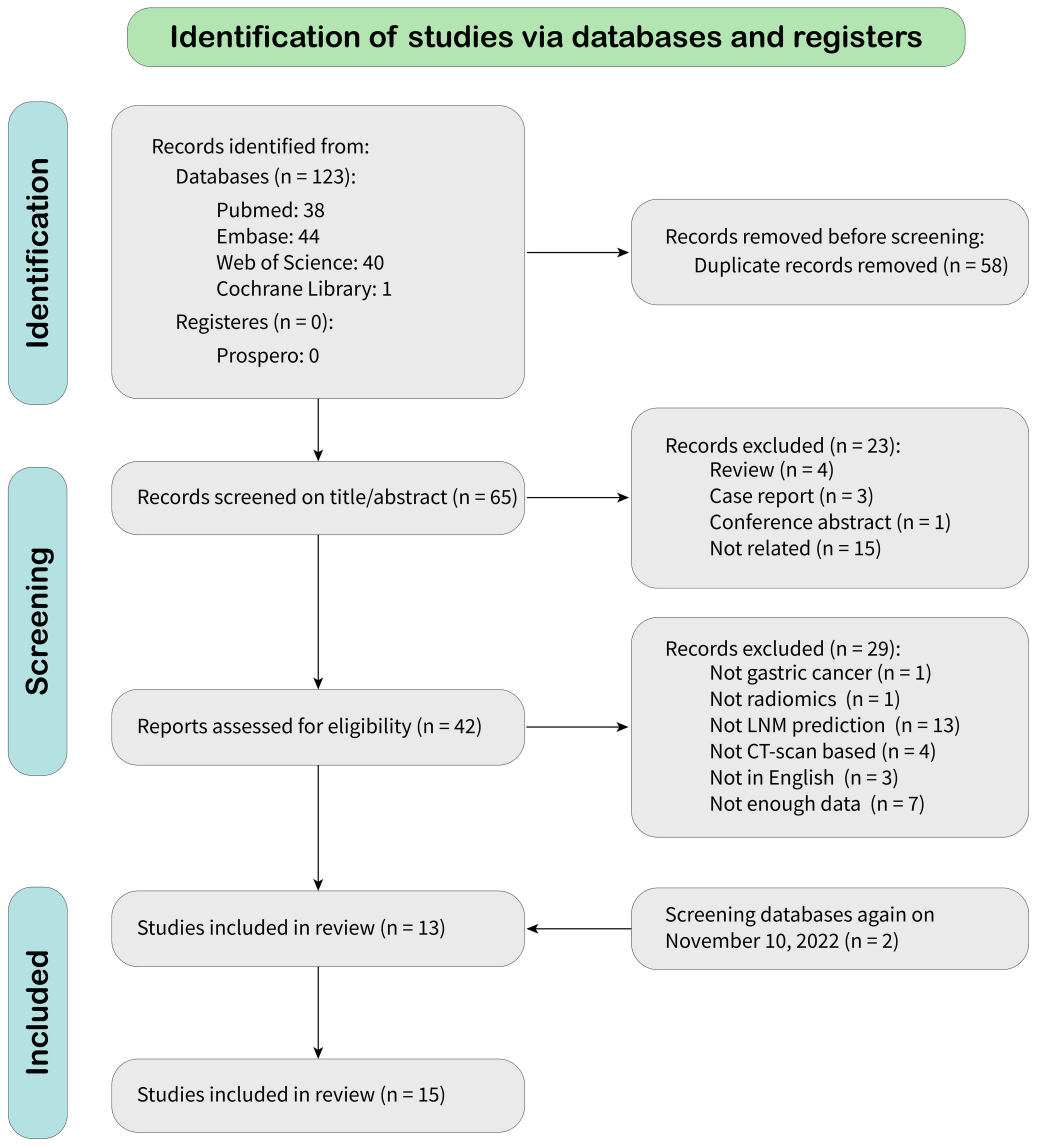
Study selection flowchart.

According to the search strategy, 123 citations were identified from databases, of which 58 were duplicates. After screening records by title and abstract, 23 were excluded because they did not meet the inclusion criteria. After a full-text review, 27 were omitted, leaving 13 articles for meta-analysis. A new literature search was repeated, and two eligible articles based on inclusion criteria were included. Finally, 15 eligible articles were selected for final meta-analysis. The detailed literature search flowchart is depicted in **Figure 1**.

### 3.2 Characteristics of included studies

Characteristics of the included studies and predictive models are shown in **Tables 1**, **2**. We enrolled 15 studies with a total number of 7,010 patients. Studies were published from July 2019 to October 2022, of which 46% (seven of 15) were published in 2021 and 2022. All study populations were from China and designed retrospectively. Only one study (25) used a prospective testing set (*n* = 112). One study (20) included patients with gastric adenocarcinoma, and the remaining studies included patients with GC. Majority of patients were male (4,935 vs. 2,075). Seven thousand ten patients were divided into a training set (*n* = 4136) and a testing set (*n* = 2874). Three studies (25, 26, 28) also used an external testing set. Eleven studies recruited patients from one center, three studies from two centers (25, 26, 28) and one study (15) from four centers. Pathological confirmation of LNM was the reference standard in all studies. Most of the studies (9/15) used a venous phase CT scan, and five used an arterial phase for lesion segmentation. One study (20) used both venous and arterial phases. Six studies used PyRadiomics for feature extraction from images.

**Table 1:** General characteristics of the included studies.

Study	No. of patients	Age	Male vs. female	Recruitment center no.	CT scan phase	Positive LNM ratio
L. Meng 2020 (15)	Total: 539 Training: 377 Internal testing: 162	58.9 vs. 59.2	372 vs. 167	Four	Venous	Total: 122/539 (22.6%) Training: 91/377 (24.1%) Internal testing: 31/162 (19.1%)
Y. Wang 2019 (16)	Total: 247 Training: 197 Internal testing: 50	60.7	167 vs. 80	One	Arterial	Total: 183/247 (74.0%) Training: 146/197 (74.1%) Internal testing: 37/50 (74.0%)
X. Gao A 2020 (17)	Total: 463 Training: 308 Internal testing: 155	46% (213/463) ≥ 60	330 vs. 133	One	Venous	Total: 83/463 (17.9%) Training: 53/308 (17.2%) Internal testing: 30/155 (19.3%)
L. Wang 2021 (18)	Total: 515 Training: 340 Internal testing: 175	58% (303/515) ≥ 60	398 vs. 117	One	Venous	Total: 64/515 (12.4%) Training: 42/340 (12.3%) Internal testing: 22/175 (12.5%)
S. Liu 2021 (19)	Total: 163 Training: 122 Internal testing: 41	64	111 vs. 52	One	Arterial	Total: 113/163 (69.3%) Training: 85/122 (69.6%) Internal testing: 28/41 (68.2%)
J. Li, 2020 (20)	Total: 204 Training: 136 Internal testing: 68	58	157 vs. 47	One	Arterial + Venous	Total: 122/204 (59.8%) Training: 84/136 (61.7%) Internal testing: 38/68 (55.8%)
X. Wang 2021 (21)	Total: 159 Training: 80 Internal testing: 79	61.7	113 vs. 46	One	Venous	Total: 39/159 (24.5%) Training: 22/80 (27.5%) Internal testing: 17/79 (21.5%)
J. Yang 2020 (22)	Total: 170 Training: 118 Internal testing: 52	61.61	112 vs. 58	One	Arterial	Total: 113/170 (66.4%) Training: 79/118 (66.9%) Internal testing: 34/52 (65.3%)
Q. Feng 2019 (23)	Total: 490 Training + Validation: 326 Internal testing: 164	61.8	363 vs. 127	One	Venous	Total: 297/490 (60.6%) Training + valid: 197/326 (60.4%) Internal testing: 100/164 (60.9%)
J. Yang 2022 (24)	Total: 170 Training: 118 Internal testing: 52	61.6 (M) vs. 58.7 (F)	112 vs. 58	One	Arterial	Total: 113/170 (66.4%) Training: 79/118 (66.9%) Internal testing: 34/52 (65.3%)
Z. Sun 2021 (25)	Total: 1618 Training: 531 External testing: 975 Prospective testing: 112	42% (680/1618) ≥ 60	1120 vs. 498	Two	Venous	Total: 978/1618 (60.4%) Training: 336/531 (63.2%) External testing: 579/975 (59.3%) Prospective testing: 63/112 (56.2%)
X. Gao B 2020 (26)	Total: 768 Training: 486 Internal testing: 240 External testing: 42	49% (383/768) ≥ 60	547 vs. 221	Two	Venous	Total: 450/768 (58.5%) Training: 281/486 (57.8%) Internal testing: 134/240 (55.8%) External testing: 35/42 (53.3%)
X. Guan 2022 (27)	Total: 347 Training: 242 Internal testing: 105	64.34	252 vs. 95	One	Arterial	Total: 212/347 (61.0%) Training: 150/242 (61.9%) Internal testing: 62/105 (59.0%)
Q. Zeng 2022 (28)	Total: 634 Training: 388 Internal testing: 167 External testing: 79	58.6 vs. 57.4 vs. 58.0	392 vs. 242	Two	Venous	Total: 214/634 (33.7%) Training: 148/388 (38.1%) Internal testing: 49/167 (29.3%) External testing: 17/79 (21.5%)
A. Zhang 2022 (29)	Total: 523 Training: 367 Internal testing: 156	59.6	389 vs. 134	One	Venous	Total: 356/523 (68.0%) Training: 247/367 (67.3%) Internal testing: 109/156 (69.8%)

**Table 2:** General characteristics of predictive models in the included studies.

Study	Segmentation	ROI	ROI software	No. of feature after/before reduction	Feature extraction software	Imaging features	Feature reduction algorithm	ICC evaluation	Modeling algorithm	Clinical factors
L. Meng 2020 (15)	Automatic	2D	NA	7/867	NA	Shape and size based, first order, texture based (GLCM, GLRLM, GLSZM, GLDM, and NGTDM)	mRMR + LASSO	Yes (> 0.75)	LR	No
Y. Wang 2019 (16)	Semi-automatic	3D	Radiomics	150/844	NA	Shape based, first order, texture based (GLCM and GLRLM), and wavelet	NA	Yes (> 0.80)	RF	CT-reported LN status
X. Gao A 2020 (17)	Manual	3D	3D slicer	6/859	PyRadiomics	First order, shape based, texture based, and wavelet	LASSO	Yes (≥ 0.85)	LASSO	CT-reported LN status
L. Wang 2021 (18)	Manual	2D	MaZda	8/352	MaZda	Geometric, texture based (GLCM, GLRLM, AR, and absolute gradient), and wavelet	LASSO + Ranker + Entropy	Yes (> 0.75)	LASSO	CT-reported LN status
S. Liu 2021 (19)	Manual	2D	Image analyzer	11/35	Image Analyzer	First order and second order (GLCM)	LASSO	No	SVM	Differentiation degree, tumor range, infiltrative growth, adipose tissue stains, morphologic type, lymphadenectasis, and CA242
J. Li, 2020 (20)	Manual	2D	GSI viewer + ITK-SNAP	2/527	ITK-SNAP	Shape based, histogram, and texture based (GLCM and GLRLM)	DCNNs	Yes (> 0.75)	ANN + SVM	CT-reported LN status
X. Wang 2021 (21)	Manual	2D	ITK-SNAP	4/273	MATLAB	Shape and size based, gray level, texture based, and wavelet	mRMR	No	LR	CT-reported LN status
J. Yang 2020 (22)	Manual	3D	ITK-SNAP	20/2394	PyRadiomics	Shape based, first order, and texture based	SFFS	No	LR	No
Q. Feng 2019 (23)	Manual	2D	ITK-SNAP	13/93	Python	First order, shape based, and texture based (GLRLM, GLCM, and GLSZM)	SVM	No	SVM	No
J. Yang 2022 (24)	Manual	3D	ITK-SNAP	∼80/2394	PyRadiomics	Shape based, first order, and texture based (GLCM, GLRLM, GLSZM, NGTDM, and GLDM)	FSBudget + Pearson correlation	No	UMvPLS	No
Z. Sun 2021 (25)	Manual	2D	ITK-SNAP	4–9/269	MATLAB	First order, shape based, and texture based (GLCM, GLRLM, and NGTDM)	LASSO + Pearson correlation	Yes	LASSO	Tumor location, size, differentiation, CEA, CA199, cT stage, and cN stage
X. Gao B 2020 (26)	Manual	3D	3D Slicer	7/859	PyRadiomics	First order, shape based, texture based, and wavelet	LASSO	Yes (≥ 0.9)	LR	CA72-4, pT stage, and CT-reported LN status
X. Guan 2022 (27)	Semi-automatic	2D	ITK-SNAP	72/-	PyRadiomics	NN features, first order, shape based, GLCM, GLSZM, GLRLM, NGTDM, and GLDM	DL ResNet50	Yes (≥ 0.8)	RF	DL features score and CT-reported LN status
Q. Zeng 2022 (28)	Manual	3D	ITK-SNAP	101/107	PyRadiomics	Shape feature, first order, and texture based	LASSO + Spearman’s correlation + CNN	Yes	SVM	Yes
A. Zhang 2022 (29)	Manual	2D	ITK-SNAP	48/851	PyRadiomics	Shape feature, histogram statistics, and second order (GLDM, GLCM, GLRLM, GLSZM, and NGTDM)	LASSO	Yes (> 0.75)	SVM	No

AR, autoregressive model; CNN, convolutional neural networks; CT, computed tomography; DCNNs, deep convolutional neural networks; GLCM, gray level co-occurrence matrix; GLDM, gray-level dependence matrix; GLRLM, gray-level run length matrix; GLRLM, gray-level run length matrix; GLSZM, gray-level size zone matrix; ICC, interclass consistency coefficient; LASSO, Least Absolute Shrinkage and Selection Operator; LN, lymph node; LR, logistic regression; mRMR, minimum redundancy maximum relevance; NA, not available; NGTDM, neighboring gray tone difference matrix; NN, neural networks; RF, random forest; ROI, region of interest; SFFS, sequential forward floating selection; SVM, support vector machine; UMvPLS, unsupervised multi-view partial least squares; 2D, two-dimensional; 3D, three-dimensional.

Open-source ITK-SNAP was the most commonly used tool for lesion segmentation (nine of 15). Twelve studies performed segmentation manually, and the other three studies performed it automatically. Most of the studies (nine of 15) delineated 2D regions of interest, and the remaining performed 3D segmentation (six of 15). Extracted features ranged from 35 to 2,394. Various methods were used in studies for image feature reduction or selection, and some used more than one method. The most often used algorithm was the Least Absolute Shrinkage and Selection Operator (LASSO) regression. Three studies (20, 27, 28) used a deep learning algorithm for feature extraction. The interclass consistency coefficient is a mathematical method for ranking the most robust features for further image analysis (30). Ten studies utilized this method for feature selection with a specific threshold. Four of them set the threshold at 0.75. Different types of features were extracted from CT scan images. Shape- and size-based (e.g., dimension), first-order (e.g., mean, maximum, and standard deviation), second-order (e.g., gray-level features), and wavelet features are the most common extracted features from images. Support vector machine (SVM) algorithm was used in five studies, logistic regression (LR) in four studies, LASSO in three studies, and random forest (RF) in two studies. One study used unsupervised multi-view partial least squares (UMvPLS) algorithm for the development of prediction models. Some studies incorporated radiomics features with clinical variables in order to establish a combined model. CT-reported LN status was the most common clinical variable used for establishing combined model.

### 3.3 Quality assessment

#### 3.3.1 RQS

The average RQS score of the included studies was 14.8, accounting for 41% of the total points. The highest RQS score was 24 points (66%), seen in only one study (25), which used a prospective dataset for model evaluation. Almost half of the studies (seven of 15) were credited between 11 and 14 points, corresponding to 30%–40% of total points. These items were not performed by studies and therefore were assigned 0 points: imaging at multiple time points, cost-effectiveness analysis, and open science and data. Details are shown in **Table 3**.

**Table 3:** Radiomics quality score and average scores of studies.

Criteria		Possible points	Average score
1	Image protocol quality	+1 if protocols are well documented +1 if public protocol is used	0.93
2	Multiple segmentations	+1 if multiple segmentations are carried out (i.e., different physicians/algorithms/software)	1
3	Phantom study	+1 if phantom study is used on all scanners	0.53
4	Multiple time points	+1 if images are collected at additional time points	0
5	Feature reduction or adjustment for multiple testing	−3 if neither measure is implemented +3 if either measure is implemented	3
6	Multivariable analysis with non-radiomics features	+1 if multivariable analysis with non-radiomics features is carried out	0.73
7	Biological correlates	+1 if phenotypic differences are demonstrated	0.2
8	Cutoff analyses	+1 if risk groups are determined by either the median, a previously published cutoff or if a continuous risk variable is reported	0.06
9	Discrimination statistics	+1 if a discrimination statistic and its statistical significance is reported (i.e., ROC curve, and AUC) +1 if a resampling method technique is also applied (i.e., bootstrapping, cross-validation)	1.73
10	Calibration statistics	+1 if a calibration statistic and its statistical significance is reported (i.e. calibration-in-the-large/slope) +1 if a resampling method technique is also applied (i.e., bootstrapping, cross-validation)	0.6
11	Prospective	+7 for prospective validation of a radiomics signature in an appropriate trial	0.46
12	Validation	−5 if validation is missing +2 if validation is based on a dataset from the same institute +3 if validation is based on a dataset from another institute +4 if validation is based on two datasets from two institutes +4 if study validates a previously published signature +5 of validation is based in three or more datasets from distinct institutes	2.33
13	Gold standard	+2 if comparison to the current gold standard is carried out	2
14	Potential clinical utility	+2 if a potential application in a clinical setting is reported	1.2
15	Cost-effectiveness analysis	+1 if the cost-effectiveness of the clinical application is reported	0
16	Open science and data	+1 if scans are open source +1 if region of interest (ROI) segmentations are open source +1 if code is open source +1 if representative segmentations and features are open source	0

#### 3.3.2 QUADAS-2

**Figure 2:**
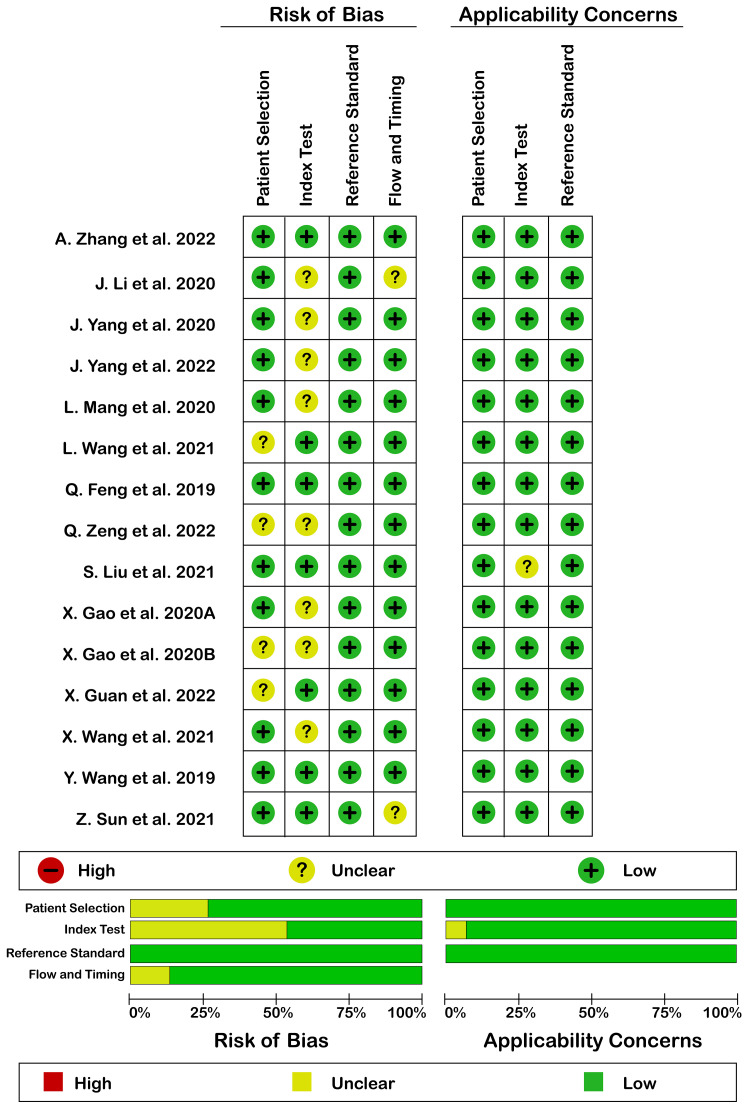
Risk of bias (left) and applicability concerns (right) of included studies using QUADAS-2 checklist.

Quality assessment according to QUADAS-2 is illustrated in **Figure 2**. Generally, quality assessment was acceptable. There was no high risk of bias or high applicability concern. The reason for the unclear risk of bias in each of the domains included: reporting consecutive or random sampling of patients in patients’ selection domain, reporting the index test interpretation without knowledge of reference standard result in the index test domain, and reporting the appropriate interval between index and reference standard test in flow and timing domain.

## 4 Data analysis

Methodologically, included studies utilized extracted CT scan features in order to establish *radiomics models* by using machine learning or deep learning mathematical algorithms. Also, a *combined model* incorporating radiomics features and clinical variables (e.g., laboratory tests and CT reported LN status) was constructed. Accordingly, we have split data analysis based on radiomics models and combined models and analyzed data separately.

In addition, included studies enrolled patients and then integrated them as a main dataset. Then, they divided the main dataset into a *training* set and *testing* set (internal testing/validation set) by a specific proportion, randomly. Training set is used for discovering and learning hidden mathematical algorithms in the dataset in order to predict the expected outcome. Finally, a prediction model is established based on those algorithms whose predictive accuracy is evaluated by an internal testing set. In order to generalize trained model, some studies utilize other datasets in addition to the main dataset and use it as a testing set (external testing set) and the predictive accuracy of the trained model is evaluated again. Therefore, in studies with various testing sets, we selected two testing sets (or cohorts) and considered them as separate studies for evaluation of predictive accuracy.

### 4.1 Radiomics model analysis

#### 4.1.1 Diagnostic accuracy

**Figure 3:**
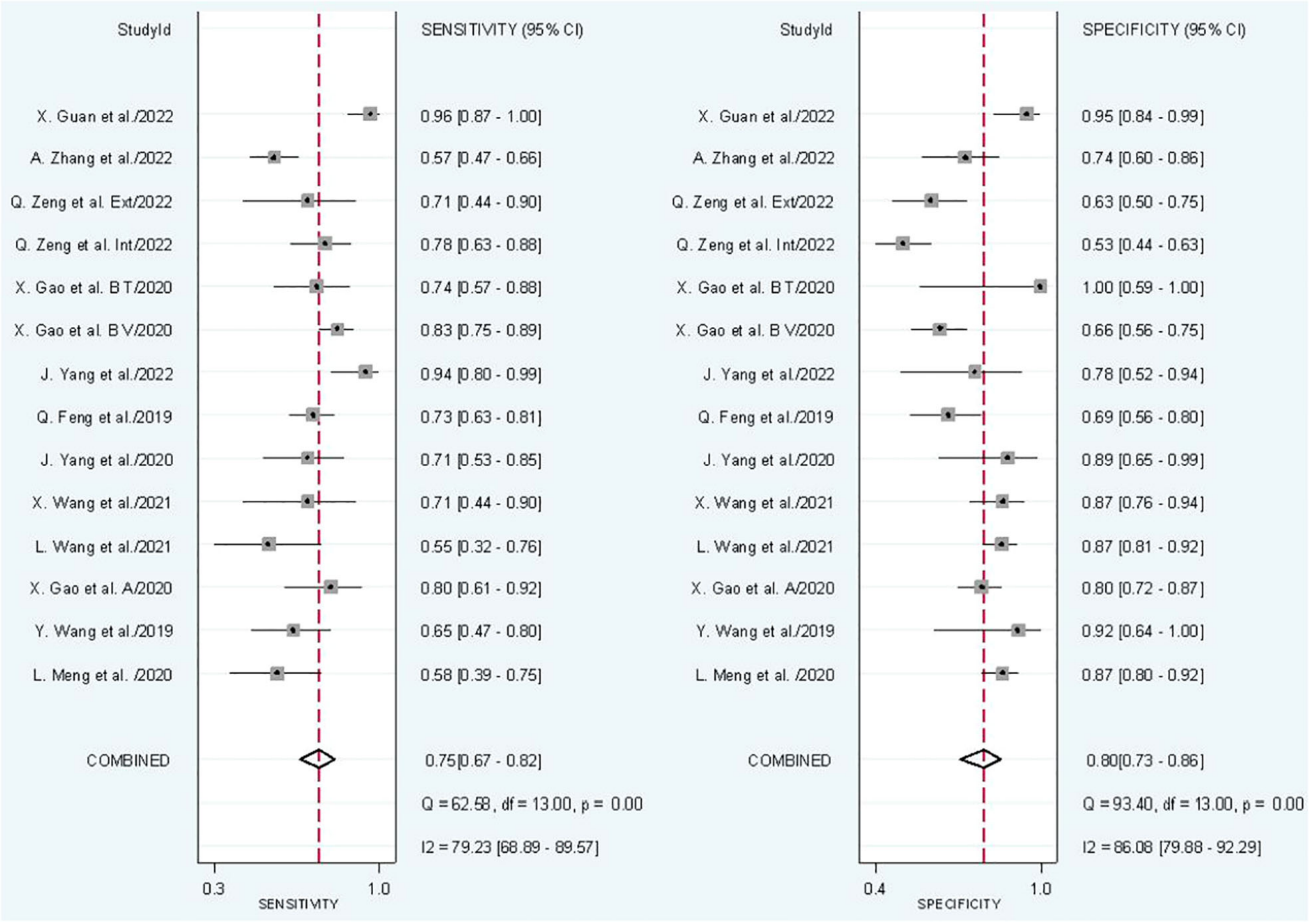
Forest plot of radiomics models.

**Figure 4:**
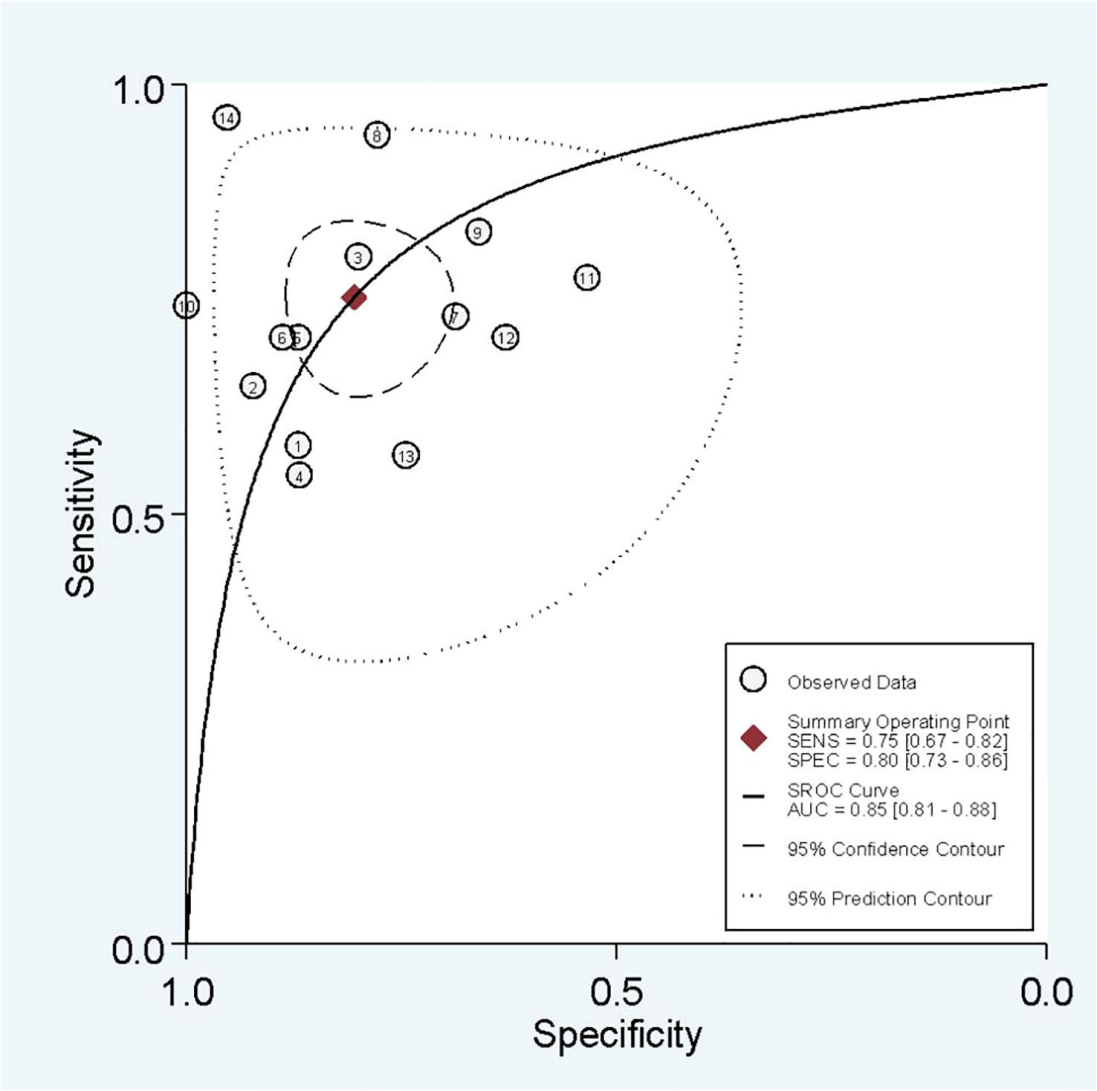
SROC of radiomics models.

In radiomics model analysis, we used 12 studies with 14 cohorts. For 14 cohorts included in radiomics model analysis, the mean value and 95% CIs of pooled sensitivity, specificity, PLR, negative likelihood ratio and DOR for radiomics models’ predictive accuracy for LNM were 0.75 [0.67, 0.82], 0.80 [0.73, 0.86], 3.9 [2.7, 5.6], 0.31 [0.23, 0.42], and 13 [7, 23], respectively. The radiomics models’ analysis showed an overall AUC of 0.85 [0.81, 0.86]. Forest plot of pooled sensitivity and specificity of radiomics models is shown in **Figure 3**, and SROC curve is illustrated in **Figure 4**.

#### 4.1.2 Heterogeneity analysis

The *I* ^2^ test showed that sensitivity (*I* ^2 = ^79.23%) and specificity (*I* ^2 = ^86.08%) both have a high heterogeneity. For threshold analysis, the Spearman’s correlation coefficient was measured as 0.046 with a *p*-value of 0.875, indicating the absence of a threshold effect.

#### 4.1.3 Subgroup analysis

Subgroup analysis was done in order to explore the heterogeneity causes (provided in **Table 4**) by comparing various study variables. Studies whose sensitivity and specificity were extracted by top left method (*n* = 6) compared to studies that did not (*n* = 8) had a higher sensitivity (0.78 vs. 0.73, *p* = 0.21) and specificity (0.82 vs. 0.79, *p* = 0.14) with a joint analysis *p*-value of 0.65. Studies that used 3D VOI (*n* = 8) compared to studies with a 2D ROI (*n* = 6) had a higher sensitivity (0.78 vs. 0.71, *p* = 0.27) but a lower specificity (0.74 vs. 0.85, *p* = 0.00) with a joint analysis *p*-value of 0.15. Arterial phase CT scan (*n* = 4) has a higher sensitivity (0.84 vs. 0.71, *p* = 0.65) and specificity (0.91 vs. 0.77, *p* = 0.90) than venous phase (*n* =10) with a joint analysis *p*-value of 0.01. Studies (*n* =3) with tumor and LNs as the ROI have a higher sensitivity (0.81 vs. 0.74, *p* = 0.06) and specificity (0.86 vs. 0.79, *p* = 0.98) than studies with only the tumoral ROI (*n* =11) with a joint analysis *p*-value of 0.49. Automatic drawn (*n* = 3) regions of interest have a higher sensitivity (0.77 vs. 0.75, *p* = 0.31) and specificity (0.91 vs. 0.76, *p* = 0.98) compared to manual segmentation (*n* = 11) with joint analysis *p*-value of 0.31.

**Table 4:** Subgroup analysis in radiomics model studies.

Variable		*n*	Sensitivity	*p*_1_	Specificity	*p*_2_	Joint model analysis		
							LRT chi^2^	*P*-value	*I* ^2^
Top left method	Yes	6	0.78 [0.69–0.88]	0.21	0.82 [0.73–0.92]	0.14	0.87	0.65	0
	No	8	0.73 [0.63–0.82]		0.79 [0.70–0.88]				
Segmentation dimension	3D (VOI)	8	0.78 [0.70–0.87]	0.27	0.74 [0.65–0.84]	0.00	3.74	0.15	47
	2D (ROI)	6	0.71 [0.60–0.83]		0.85 [0.78–0.92]				
Phase	Arterial	4	0.84 [0.75–0.93]	0.65	0.91 [0.84–0.99]	0.90	9.37	0.01	54
	Venous	10	0.71 [0.62–0.79]		0.77 [0.69–0.84]				
Tumoral or nodal segmentation	Tumoral	11	0.74 [0.66–0.82]	0.06	0.79 [0.71–0.86]	0.05	1.42	0.49	0
	Tumoral and nodal	3	0.81 [0.67–0.95]		0.86 [0.74–0.98]				
Automatic or manual segmentation	Automatic	3	0.77 [0.62–0.92]	0.31	0.91 [0.84–0.98]	0.98	5.52	0.06	64
	Manual	11	0.75 [0.66–0.83]		0.76 [0.69–0.83]				

p, p-value; ROI, region of interest; VOI, volume of interest.

#### 4.1.4 Publication bias

**Figure 5:**
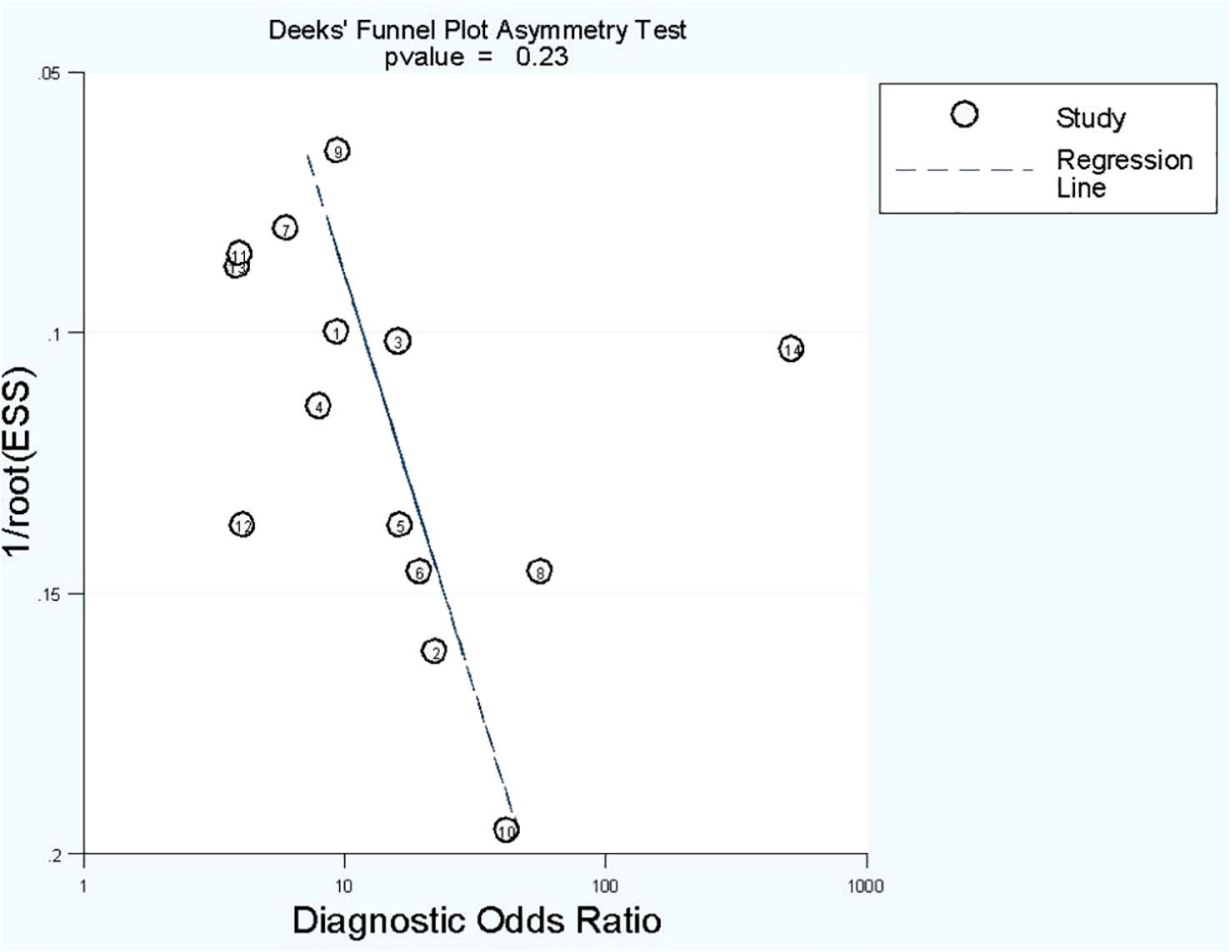
Funnel plot of publication bias based on Deek’s asymmetry test in radiomics model studies.

No publication bias was found in radiomics model studies based on deeks funnel plot (*p* = 0.23) (**Figure 5**).

### 4.2 Combined model analysis

#### 4.2.1 Diagnostic accuracy

**Figure 6:**
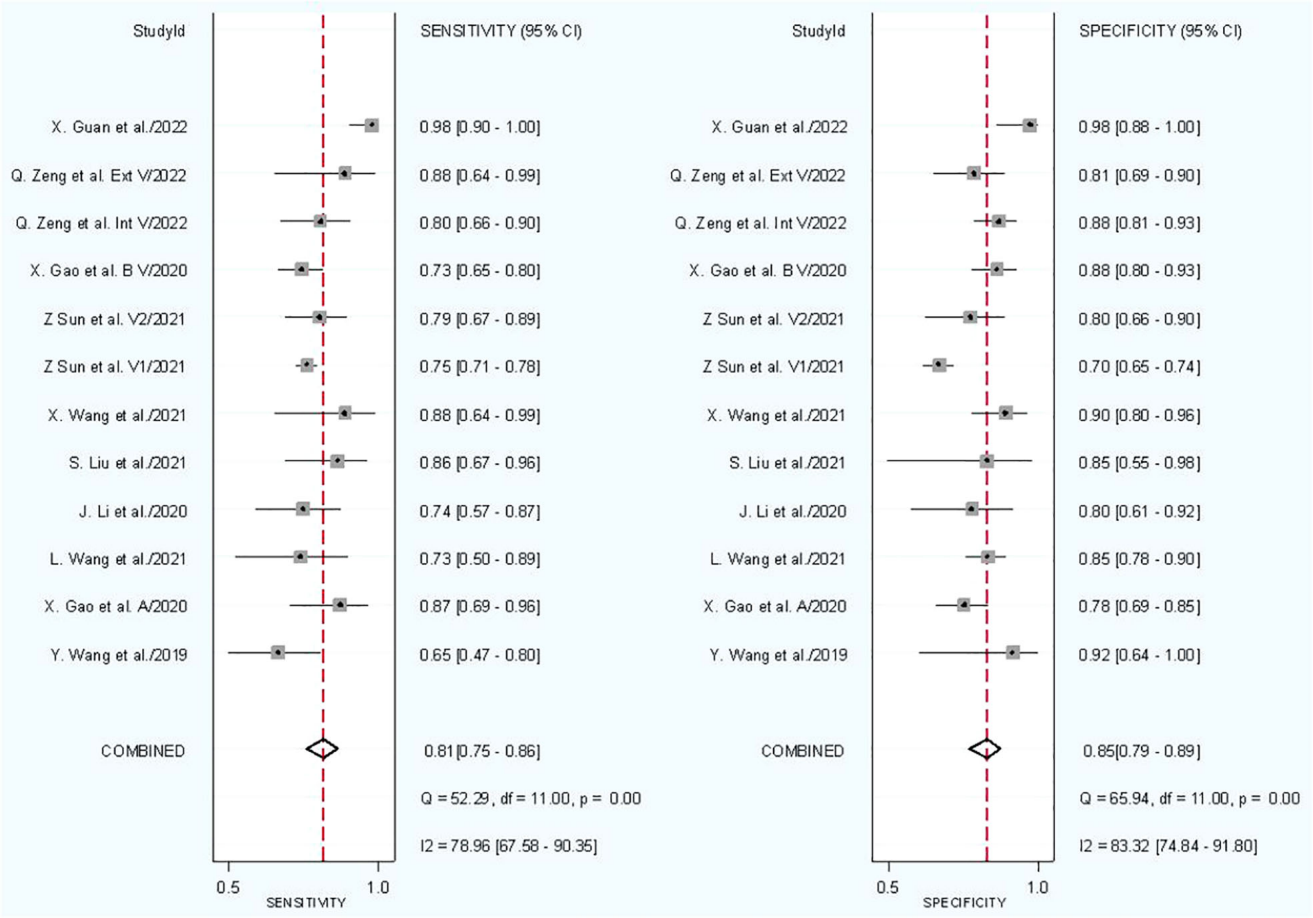
Forest plot for combined models.

**Figure 7:**
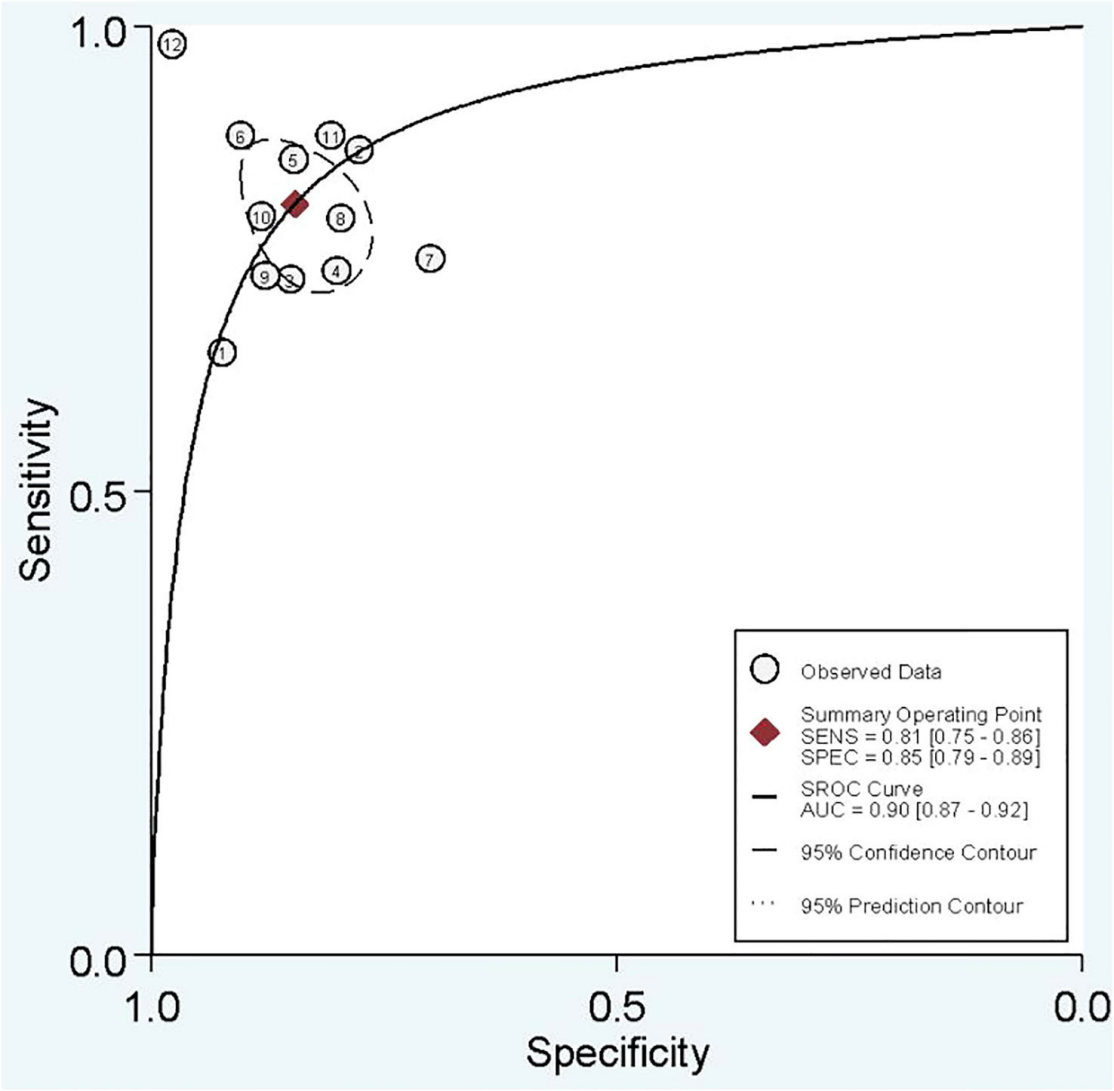
SROC of combined model studies.

In combined model analysis, we used 10 studies with 12 cohorts. For 12 cohorts included in radiomics nomogram analysis, the mean value and 95% CIs of pooled sensitivity, specificity, PLR, negative likelihood ratio and DOR for radiomics nomogram predictive accuracy for LNM were 0.81 [0.75, 0.86], 0.85 [0.79,0.89], 5.2 [3.7, 7.4], 0.23 [0.16, 0.31], and 23 [13,42] respectively. The radiomics models’ analysis showed an overall AUC of 0.90 [0.87, 0.92]. Forest plot of pooled sensitivity and specificity of combined models is shown in **Figure 6**, and the SROC curve is illustrated in **Figure 7**.

#### 4.2.2 Heterogeneity analysis

The *I* ^2^ test showed that sensitivity (*I* ^2 = ^78.96%) and specificity (*I* ^2 = ^83.32%) both have a high heterogeneity. For threshold analysis, the Spearman’s correlation coefficient was measured as −0.081 with a *p*-value of 0.803, indicating the absence of a threshold effect.

#### 4.2.3 Subgroup analysis

Subgroup analysis was done in order to explore the heterogeneity causes (provided in **Table 5**) by comparing various study variables. Studies whose sensitivity and specificity were extracted by top left method (*n* = 5) compared to studies which did not (*n* = 7) had a higher sensitivity (0.83 vs. 0.79, *p* = 0.05) and lower specificity (0.83 vs. 0.86, *p* = 0.00) with a joint analysis *p*-value of 0.43. Studies that used 2D VOI (*n* = 7) compared to studies with 3D ROI (*n* = 5) had a higher sensitivity (0.84 vs. 0.75, *p* = 0.00) and specificity (0.85 vs. 0.84, *p* = 0.00) with a joint analysis p value of 0.20. Arterial phase CT scan (*n* = 3) have a higher sensitivity (0.86 vs. 0.80, *p* = 0.20) and specificity (0.94 vs. 0.83, *p* = 0.91) than venous phase (*n* = 8) with a joint analysis p value of 0.14. Studies (*n* = 1) with tumor and LNs as the ROI have a higher sensitivity (0.89 vs. 0.80, *p* = 0.36) and specificity (0.91 vs. 0.84, *p* = 0.07) than studies with only tumoral ROI (*n* =11), with a joint analysis *p*-value of 0.56. Automatic drawn (*n* = 2) ROI has a higher sensitivity (0.85 vs. 0.80, *p* = 0.28) and specificity (0.96 vs. 0.83, *p* = 0.20) compared to manual segmentation (*n* = 10) with joint analysis *p*-value of 0.07.

**Table 5:** Subgroup analysis in combined model studies.

Variable		*n*	Sensitivity	*p*_1_	Specificity	*p*_2_	Joint model analysis		
							LRT chi^2^	*P*-value	*I* ^2^
Top left method	Yes	5	0.83 [0.75–0.90]	0.05	0.83 [0.76–0.90]	0.00	1.69	0.43	0
	No	7	0.79 [0.70–0.87]		0.86 [0.80–0.91]				
Segmentation dimension	3D (VOI)	5	0.75 [0.67–0.83]	0.00	0.84 [0.77–0.91]	0.00	3.26	0.20	39
	2D (ROI)	7	0.84 [0.78–0.89]		0.85 [0.78–0.91]				
Phase	Arterial	3	0.86 [0.76–0.95]	0.20	0.94 [0.87–1.00]	0.91	3.92	0.14	49
	Venous	8	0.80 [0.73–0.87]		0.83 [0.78–0.88]				
Tumoral or nodal segmentation	Tumoral	11	0.80 [0.75–0.86]	0.36	0.84 [0.79–0.89]	0.07	1.16	0.56	0
	Tumoral and nodal	1	0.89 [0.72–1.00]		0.91 [0.80–1.00]				
Automatic or manual segmentation	Automatic	2	0.85 [0.74–0.96]	0.28	0.96 [0.91–1.00]	0.20	5.46	0.07	63
	Manual	10	0.80 [0.74–0.86]		0.83 [0.78–0.87]				

p, p-value; ROI, region of interest; VOI, volume of interest.

#### 4.2.4 Publication bias

**Figure 8:**
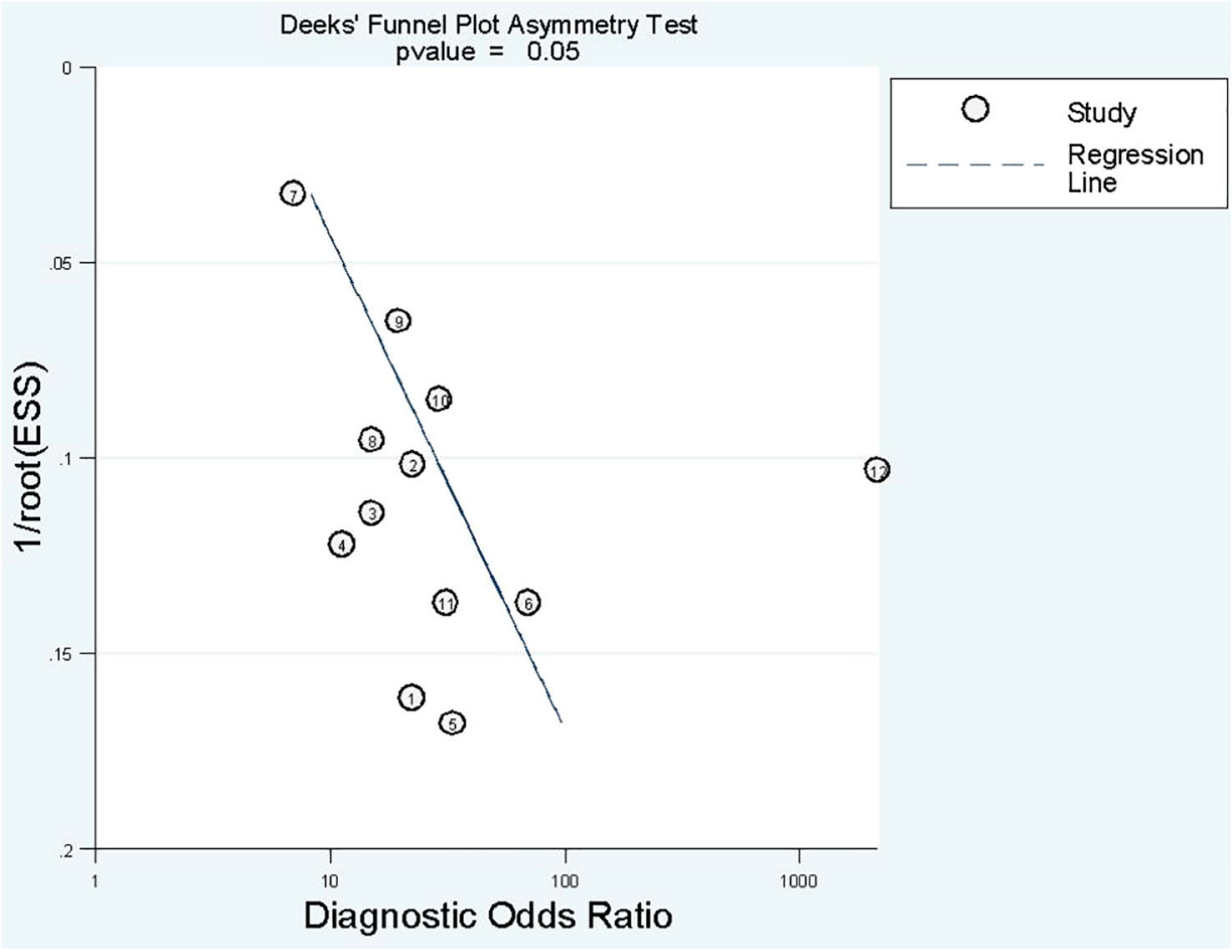
Funnel plot of publication bias based on Deek’s asymmetry test in combined models.

Deek’s funnel plot has shown a publication bias in combined model studies (*p* = 0.05) (**Figure 8**). Therefore, we performed sensitivity analysis.

#### 4.2.5 Sensitivity analysis

We eliminated included cohorts in combined model analysis one by one, and the changes were observed. Eliminating the study by Z. Sun et al. (25). showed that increased *p*-value significantly, thus, reducing publication bias (**Table 6**). It can be explained by the large number of participants in the study. Also, the top left method used for calculation of sensitivity and specificity is also can be a reason.

**Table 6:** Results of sensitivity analysis.

Study eliminated	Sensitivity	*I* ^2^	Specificity	*I* ^2^	PLR	NLR	DOR	AUC	Deek’s *P*-value
Y. Wang 2019 (16)	0.82	81.84	0.85	85.57	5.3	0.21	25	0.90	0.06
X. Gao A 2020 (17)	0.81	81.51	0.85	85.36	5.5	0.23	24	0.90	0.07
L. Wang 2021 (18)	0.81	81.49	0.85	84.48	5.3	0.22	24	0.90	0.06
J. Li, 2020 (20)	0.82	82.56	0.85	85.63	5.4	0.22	25	0.90	0.05
S. Liu 2021 (19)	0.81	80.07	0.84	84.56	5.2	0.23	23	0.89	0.06
X. Wang 2021 (21)	0.80	77.90	0.84	82.60	5.0	0.23	21	0.89	0.08
Z. Sun 2021 (25) Validation set 1	0.82	59.83	0.86	45.90	5.7	0.22	26	0.91	0.76
Z. Sun 2021 (25) Validation set 2	0.81	82.18	0.85	85.45	5.4	0.22	25	0.90	0.06
X. Gao B 2020 (26)	0.82	82.80	0.84	83.95	5.1	0.21	24	0.89	0.07
Q. Zeng 2022 (28) Internal validation set	0.81	81.10	0.84	83.30	5.1	0.22	23	0.90	0.07
Q. Zeng 2022 (28) External validation set	0.81	80.90	0.85	85.18	5.4	0.23	24	0.90	0.07
X. Guan 2022 (27)	0.76	51.91	0.83	78.08	4.5	0.29	16	0.78	0.00
Z. Sun 2021 (25) Validation set 1 and 2	0.82	63.98	0.86	50.65	6.0	0.21	29	0.91	0.80
Q. Zeng 2022 (28) External and internal validation set	0.81	83.01	0.85	85.36	5.3	0.23	23	0.90	0.09

AUC, area under the ROC curve; DOR, diagnostic odds ratio; NLR, negative likelihood ratio; PLR, positive likelihood ratio.

## 5 Discussion

This meta-analysis investigated the utility of radiomics-based models based on CT scan images for the prediction of LNM occurrence in GC patients preoperatively. Our analysis showed that radiomics-based models have a promising potential for the prediction of positive LNM in GC. However, the relatively low quality of performing and reporting of radiomics studies in GC is currently suboptimal to allow radiomics to be widely adopted in clinical applications. Nevertheless, it has become evident that radiomics approaches have a promising role in the discrimination of target lesion classes in GC patients at high risk for LNM. Thus, if studies follow the same methodological guidelines more strictly and also use large and comprehensive datasets from several centers, we may create an excellent opportunity for radiomics application for more tailored therapies, thus reaching better clinical outcomes.

Recently, the radiomics approach as a non-invasive diagnostic tool offered a new perception for clinicians in disease management, especially in the field of oncology. Therefore, a growing number of papers investigated radiomics applicability in cancers of different organs such as gastrointestinal, respiratory, neurological, and breast (30). Focusing on the prediction of LNM in cancers, a previous meta-analysis of 12 studies (793 patients) by Longchao Li et al. (31) culminates that MRI-based radiomics models have a promising diagnostic accuracy in cervical cancer with a pooled sensitivity, specificity, and AUC of 80%, 76%, and 0.83, respectively. Limitations of study conduction reported by authors were a limited number of subjects and recruitment centers with a high rate of heterogeneity, especially different magnetic resonance protocols and imaging equipment technology among studies. Another meta-analysis by Jing Zhang et al. (32) with 13 studies (1,618 patients) examined LNM presence based on machine learning–based radiomics of dynamic contrast-enhanced magnetic resonance imaging (DCE-MRI) in breast cancer. Their analysis showed that the pooled sensitivity, specificity, and AUC were 82%, 83%, and 0.89, which offers a good discrimination ability of radiomics models. The authors reported that the small number of patients, significant heterogeneity, and low-quality assessment scores were the major limitations of the studies.

Generally, radiomics studies select patients and consider them as a main dataset for model construction. The main dataset is randomly divided into training and internal testing sets. First, the radiomics model learns the unseen mathematical pattern and structure of the dataset from the training set. The developed model needs to be evaluated and tested for its performance and generalizability. There are two types of testing datasets: internal and external testing sets. Internal testing is derived from the same dataset from which the training dataset was taken. The second type is external testing, which is selected from a different institution and region. Therefore, the developed model uses testing sets for performance evaluation. Using external testing helps radiomics approach to be more generalized and comprehensive in order to have a role in clinical practice. Three studies (three of 15) used an external testing set. Furthermore, we can integrate radiomics models established by imaging features with other clinical data and develop a new model called the “combined model” (30).

In the current study, we separate analyses based on the radiomics model and combined model separately. Some studies used both radiomics model and combined model. Others used only one of them. The sensitivity, specificity, and AUC of the radiomics model were approximately 75%, 80%, and 0.85, indicating good performance. It is evident that pathological confirmation of LNM, which is the reference standard of included studies, is determined postoperatively. Thus, if we need to tailor therapies regarding LNs status, it is better to determine it before surgery. Radiomics models have an excellent ability to forecast three-fourths of LNM-positive patients preoperatively without unnecessary invasive interventions. Moreover, a specificity of 80% gives us a good level of certainty that positive LNM patients predicted by radiomics model need therapy optimization. A combined model integrating radiomics features and clinical variables, is associated with an improvement in predictive ability. Overall sensitivity, specificity, and AUC of 81%, 85%, and 0.90 show that an adjunct of clinical variables to radiomics features can help us to improve predictive capacity. Taken together, we conclude that incorporating radiomics features with other clinical variables provides better diagnostic performance.

Despite this, an apparent heterogeneity was found among the studies. Thus, we explored possible heterogeneity sources using subgroup analysis to pave the way for upcoming studies. Spearman’s correlation coefficients were not the heterogeneity sources. We were concerned about the difference between studies with calculated top left method and studies which did not. Results showed that the calculated top left point had a slightly better performance. CT scan phase differences were also explored, and results showed that the arterial phase has a better outcome than the venous phase in both radiomics and combined models. Image segmentation is a crucial process in radiomics approach, since radiomics features will be extracted from the delineated areas (33).

3D segmentation had only a better sensitivity in radiomics models. Otherwise, 2D segmentation had an overall higher value than 3D segmentation. Surprisingly, selecting the largest imaging plane for segmentation showed that 2D segmentation not only has better results but also it is less time consuming and simple. Segmentation of the tumoral area has shown to have a better predictive performance compared to tumoral and nodal areas in both radiomics and combined models. Although manual segmentation of the ROI is preferred in the majority of studies, automatic and semi-automatic segmentations discriminate better than manual segmentation in both radiomics and combined models.

Despite the promising results in this study, the RQS scores of studies were low to moderate ranging from 11 to 24 of 36 possible scores. Only three studies tested the model’s performance externally. Of note, only one study used a prospective dataset (25). QUADAS-2 quality assessment revealed some issues to be optimized in upcoming papers, for example, mentioning the consecutive or random sampling of patients, reporting the blindness of readers to the pathological status of samples, and reporting the interval between the index test and reference test.

## 6 Limitation

This review highlights some limitations in studies as reflected by methodological assessment. We had to exclude a number of studies that achieved the inclusion criteria but did not have enough data to analyze, which indicates a pitfall in reporting results. Studies acquired a significant heterogeneity score, which was similar to previous diagnostic radiomics meta-analyses (31, 32).

Also, included studies presented a relatively small and wide range of patient numbers. The majority of datasets were selected retrospectively, which can contribute to selection bias. In addition, patient recruitment from one center restricted results from being generalized and reproducible. Four studies (four of 15) used more than one center for patient selection. Additionally, studies used different CT scanning protocols. We only could overcome the arterial and venous phase differences by subgroup analysis but still the high heterogeneity of CT scanning protocols and techniques between studies could not be overcome by subgrouping. Moreover, in most studies, the GC stage and LN station were not considered in image analysis and modeling. Therefore, the extracted and selected features are different, which obviously affects the performance of models and also leads to inter-study heterogeneity. In addition, the segmentation methods and software used in studies can affect models. Taken together, the main obstacle in studies was the heterogeneities in study methodologies. Therefore, it shows the necessity of establishing a unified standard and guideline for radiomics accomplishment, and more importantly, future explorations should adhere to the standards.

## 7 Conclusion

Our analysis demonstrated that the CT scan–based radiomics approach seems promising for predicting LNM in GC patients before surgery and has an excellent diagnostic accuracy for surgery planning and personalized therapy. Nevertheless, high heterogeneity of studies indicates the necessity of a unified guideline for radiomics conduction in upcoming research. Therefore, so far it is crucial to consider radiomics limitations in clinical application.

## Data availability statement

The raw data supporting the conclusions of this article will be made available by the authors, without undue reservation.

## Author contributions

ZH and LA designed the study strategy. ZH and FP searches the database and selected the studies. ZH and PT extracted the data and evaluated the studies quality. PT and FP did the analysis. ZH wrote the manuscript and edited by LA, BB and PT. LA and BB supervised the work. All authors contributed to the article and approved the submitted version.
